# Automated High-Content Assay for Compounds Selectively Toxic to *Trypanosoma cruzi* in a Myoblastic Cell Line

**DOI:** 10.1371/journal.pntd.0003493

**Published:** 2015-01-23

**Authors:** Julio Alonso-Padilla, Ignacio Cotillo, Jesús L. Presa, Juan Cantizani, Imanol Peña, Ana I. Bardera, Jose J. Martín, Ana Rodriguez

**Affiliations:** 1 Parasitology Division, Department of Microbiology, New York University School of Medicine, New York, New York, United States of America; 2 Diseases of the Developing World, GlaxoSmithKline, Tres Cantos, Madrid, Spain; 3 Molecular Discovery Research, GlaxoSmithKline, Tres Cantos, Madrid, Spain; Institut Pasteur, FRANCE

## Abstract

**Background:**

Chagas disease, caused by the protozoan parasite *Trypanosoma cruzi*, represents a very important public health problem in Latin America where it is endemic. Although mostly asymptomatic at its initial stage, after the disease becomes chronic, about a third of the infected patients progress to a potentially fatal outcome due to severe damage of heart and gut tissues. There is an urgent need for new drugs against Chagas disease since there are only two drugs available, benznidazole and nifurtimox, and both show toxic side effects and variable efficacy against the chronic stage of the disease.

**Methodology/Principal Findings:**

Genetically engineered parasitic strains are used for high throughput screening (HTS) of large chemical collections in the search for new anti-parasitic compounds. These assays, although successful, are limited to reporter transgenic parasites and do not cover the wide *T. cruzi* genetic background. With the aim to contribute to the early drug discovery process against Chagas disease we have developed an automated image-based 384-well plate HTS assay for *T. cruzi* amastigote replication in a rat myoblast host cell line. An image analysis script was designed to inform on three outputs: total number of host cells, ratio of *T. cruzi* amastigotes per cell and percentage of infected cells, which respectively provides one host cell toxicity and two *T. cruzi* toxicity readouts. The assay was statistically robust (Z´ values >0.6) and was validated against a series of known anti-trypanosomatid drugs.

**Conclusions/Significance:**

We have established a highly reproducible, high content HTS assay for screening of chemical compounds against *T. cruzi* infection of myoblasts that is amenable for use with any *T. cruzi* strain capable of *in vitro* infection. Our visual assay informs on both anti-parasitic and host cell toxicity readouts in a single experiment, allowing the direct identification of compounds selectively targeted to the parasite.

## Author Summary

Chagas disease is a zoonosis caused by the protozoan parasite *Trypanosoma cruzi*. Endemic to Central and South America, it affects over 10 million people and many more live in risk transmission areas. Although mostly asymptomatic at its initial acute stage in humans, damages can occur over the years in many tissues such as *T. cruzi*-hosting heart and digestive track. For over 40 years the chemotherapy of Chagas disease has relied on the use of two drugs, benznidazole and nifurtimox, though both are known to lead to severe side effects which often prompt to the discontinuation of the treatments. Despite having good efficacy against the acute stage of infection, its efficacy against the chronic stage is under question. Therefore, there is an urgent unmet need of new anti-*T. cruzi* drugs. Several efforts have been made in the last years to establish reliable high throughput cell based *in vitro* assays to be used for drug discovery against Chagas. With the aim to contribute to this field here we describe the development of a new automated image-based assay to identify new compounds against *T. cruzi* that has been set up using the myoblastic rat cell line H9c2 as cell-cycling amastigotes hosting cells.

## Introduction

Chagas disease, classified by the WHO as a neglected tropical disease, is a zoonosis caused by the Kineoplastid protozoan parasite *Trypanosoma cruzi*. It is endemic to Central and South America where it stands as a major public health problem [1]. Migratory population movements between endemic and non-endemic regions during the past decades have globalized Chagas impact, which nowadays represents a public health issue in several non-endemic countries [1, 2]. It is estimated that 12,000 people die annually of Chagas, there are 10 million people currently infected, and 100 million live in risk-transmission areas [3].

The disease progresses over three phases. First, an early acute stage barely spanning over a few weeks during which parasitemia is detectable. Though mostly asymptomatic, it can cause the death of children and immune-suppressed patients. A second indeterminate stage follows that can last over several years (10 to 20), where the parasite presence is hardly detected and no symptoms are observed. Finally, in about 30% of the chronically infected patients, the infection can lead to severe failures of heart and gastrointestinal tract functions that ultimately cause death [4].

There is no effective human vaccine against Chagas disease currently available and its development may entail potentially insurmountable hurdles [2, 5]. The two available drugs against the disease are benznidazole (BNZ; Abarax, laboratorio ELEA) and nifurtimox (NFX; Lampit, Bayer Healthcare), which although have a good efficacy against the short-lasting, mostly asymptomatic acute phase of the disease, its activity against the life-threatening chronic phase is still under study [2, 6]. Both BNZ and NFX have toxic side-effects that often cause premature discontinuation of the treatments. An important difficulty in Chagas drug development is that success in treatment is difficult to evaluate since this is a silent disease that shows its symptomatology decades after the infection and the presence of parasites in the blood is at the limit of detection during the chronic stage [4]. Recently, the promising results achieved with the repurposed anti-fungal azoles (posaconazole and the ravuconazole derivative E1224; [7, 8]) took them into clinical trials, but unfortunately the outcome of both has been disappointing [9–10]. With such outlook, high expectations are put in the discovery of less toxic drug entities that are active against the chronic stage of the infection. As it happens with many other neglected tropical diseases, there is an urgent unmet requirement of new chemotherapies that should widely improve the performance of current treatments [6, 11, 12].

In the field of drug discovery against infectious diseases, high-content screening approaches are being increasingly used since they allow the acquisition and analysis of highly informative data from cell-culture based events [13–14]. Several assays suitable for high throughput screening of chemical collections, including image-based assays and an algorithm to assess anti-*T. cruzi* drug inhibition, have been described for early drug discovery in Chagas disease [11, 15–18]. With the aim to contribute to the drug search process against Chagas, here we describe the development of a phenotypic assay to identify anti-*T. cruzi* compounds, that allows high throughput with excellent reproducibility. The assay has been set up on a 384 well plate format with an Opera high-content microscope (Perkin-Elmer), which can be automated to sustain a throughput adequate for both primary screening of compounds or secondary hit qualifier. Based in microscopic image analysis, the developed assay can be used to test any parasite strain that is adapted for infection *in vitro* (including non-engineered strains and clinically relevant specimens), and since host-cell and parasite drug effects are simultaneously assessed, it provides specific anti-parasitic readouts and host cell toxicity information in a single experiment. In order to achieve a biologically relevant environment, the assay was set up using the rat heart derived cell line H9c2 as host cells.

## Methods

### Parasite and mammalian cell cultures

LLC-MK2 (green monkey kidney epithelial cells) and H9c2 (rat cardiomyocytes) cell lines were cultivated in DMEM (Life-Technologies) supplemented with 10% FBS (Biowest, USA), 100 U/ml penicillin (Sigma-Aldrich), 100 μg/ml streptomycin (Sigma-Aldrich), and 4 mM or 2 mM L-glutamine (Sigma-Aldrich), respectively. Both cell lines were purchased at the European Collection of Cell Cultures (ECACC, Salisbury, UK) and were grown at 37°C, 5% CO_2_ and >95% humidity. H9c2 cells were cultured in roller flasks (800 cm^2^ growing surfaces; Corning Inc., NY, USA). A single roller supplied cells to seed at least seven T225 flasks at the assay day. The DMEM formulation for the assay lacked phenol red (Life-Technologies reference 31053) and was supplemented with 2% FBS, 100 U/ml penicillin, 100 μg/ml streptomycine, 2 mM L-Glutamine, 1 mM sodium-pyruvate (Life-Technologies), and 25 mM HEPES (Life-Technologies) [15].

*T. cruzi* Tulahuen strain parasites expressing β-galactosidase were kindly provided by Dr. Buckner (University of Washington, Seattle, USA; [19]) and maintained in culture by weekly infection of LLC-MK2 cells in the same DMEM formulation used for cell growth, but supplemented with 2% FBS instead of the 10% FBS added to the cell lines maintenance medium. Trypomastigote forms were obtained from the supernatants of LLC-MK2 infected cultures harvested between days 5 and 8 of infection as described elsewhere [15]. They were used to maintain the cycle and to infect H9c2 monolayers.

### Preparation of compounds

The compounds used to set up the assay were selected upon literature searches based in their previously described anti-trypanosomatid activity (see Table 1 for details) or their presence in current clinical trials against *T. cruzi* [12]. Those compounds not available in GSK chemical collection were purchased from Sigma-Aldrich except the following: amiodarone (Pfizer), cloroxylenol derivative CX1 (Chembridge), dihydroergocristine mesylate (Tocris Bioscience), hydrazide derivative PCH1 (InterBioScreen), LP10 (ChemDiv), loperamide (Enamine), posaconazole (Sheckchem.com), pubchem 1473168 and pubchem 3812524 (Bionet), and terconazole (AKSCI-USA). Compounds were pre-dispensed into the plates with an Echo 555 instrument (Labcyte; 250 nl per well) in a 3-fold dilution row-pattern to get eleven concentration points for each compound. Compound concentrations in the assay ranged from 10^2^ to 1.6×10^-3^ μM except posaconazole that ranged from 1 to 1.6×10^-5^ μM. Upon preparation, the plates were stored at -20°C until being assayed. Whole ranges were used to determine host-cell toxicity (TC_50_) and anti-parasitic efficacy (IC_50_) values.

**Table 1:** List of compounds used to validate the assay.

**Compound**	**TC_50_ (μM)**	**IC_50_^a^ (μM)**	**IC_50_^b^ (μM)**	**S.I.^c^**	**Ref^d^**	**Reported IC_50_^a^ (μM)**	**Anti-*T. cruzi* HTS results^#^**
Allopurinol	>100 ± nd^e^	1.36 ± 0.59	2.28 ± 1.47	>73.53	[20]	12	4.25
Amiodarone	9.43 ± 5.00	1.29 ± 0.39	4.61 ± 1.42	7.31	[17]	0.8	4.57
Azelastine	35.11 ± 4.23	0.93 ± 0.22	1.77 ± 0.28	37.75	[17]	1	0.4
Benznidazole	>100 ± nd	1.43 ± 0.21	5.02 ± 2.41	>69.93	[19]	1.5	0.83
CX1	14.63 ± 3.64	0.01 ± 0.01	3×10^-3^ ± 2×10^-3^	>1.46×10^3^	[15]	0.023	0.055
Cycloheximide	2.69 ± 0.68	9×10^-3^ ± 2×10^-3^	1.6×10^-2^ ± 6×10^-3^	298.88	[17]	<0.4	2.3×10^-3^
Dihydroergocristine mesylate	18.01 ± 2.10	7.10 ± 2.33	15.33 ± 6.54	2.54	[17]	5	5.35
Eflornithine	>100 ± nd	>100 ± nd	>100 ± nd	nd	[21]	-	-
Fenarimol	72.90 ± 17.90	0.29 ± 0.10	0.38 ± 0.07	251.38	[22]	-	0.08
Furazolidone	74.13 ± 44.81	0.28 ± 0.05	0.73 ± 0.23	264.75	[17]	<0.4	0.07
PCH1	>100 ± nd	0.12 ± 0.02	0.16 ± 0.03	>833.33	[15]	0.054	0.088
Lonidamine	>100 ± nd	1.96 ± 0.76	2.23 ± 0.72	>51.00	[23]	-	72.7
Loperamide	14.10 ± 3.54	0.73 ± 0.06	0.90 ± 0.03	19.32	[17]	3	2.3
LP10	>100 ± nd	0.67 ± 0.27	0.82 ± 0.35	>149.3	[24]	-	0.025
Nifurtimox	>100 ± nd	0.35 ± 0.05	1.39 ± 0.36	>285.71	[25]	7.7	0.27
Posaconazole	>100 ± nd	2×10^-4^ ± 3.4×10^-6^	3×10^-4^ ± 8×10^-5^	>5×10^5^	[26]	3×10^-4^ *	2×10^-3^
Pubchem 1473168	>100 ± nd	0.22 ± 0.07	0.41 ± 0.09	>454.55	[27]	<0.4	0.24
Pubchem 3812524	>100 ± nd	0.09 ± 0.02	0.27 ± 0.09	>1.1×10^3^	[27]	0.2	0.19
Quinacrine	2.51 ± 0.62	1.07 ± 0.19	3.37 ± 0.75	2.34	[28]	-	1.38
Terconazole	14.43 ± 2.84	0.01 ± 8×10^-4^	9×10^-3^± 1×10^-3^	1.4×10^3^	[17]	<0.4	0.01

Their host cell toxicity (TC_50_) and anti-*T. cruzi* (IC_50_ for the ‘Am/Cell’ and ‘%Infectivity’ assay outputs) mean values of three independent experiments plus their sd are shown (mean ± sd).

^a^IC_50_ values for the ‘Am/Cell’ output;

^b^IC_50_ values for the ‘%Infected’ output;

^c^ratio between the mean TC_50_ values and their corresponding ‘Am/Cell’ IC_50_ measurement.

^d^literature reference;

^e^nd, not determined;

^#^values obtained at the anti-Chagas HTS campaign recently performed at GSK (Peña et al., manuscript in preparation);

*not actually an IC_50_ value but the minimal compound dose to eradicate T. cruzi amastigotes in Vero cells [26].

Control wells used to determine a 100% parasitic growth (full 6^th^ column of each plate) were left untreated, whereas not infected cells were added in the control wells used as 0% parasite growth (or 100% parasite growth inhibition; full 18^th^ column of each plate).

### Infection of H9c2 cells by *T. cruzi* parasites

H9c2 cells were seeded in T-225 flasks (5 × 10^6^ cells/flask, 225 cm^2^ culture surface; Corning Inc., NY, USA) in DMEM-10% FBS for 4 h to allow attachment. Cells were then washed once with PBS before infection. *T. cruzi* trypomastigotes, collected at days 5 to 8 after infection, from LLC-MK2 parasite infected cultures, were allowed to swim out for 4 hours at 37°C from a centrifuged pellet (2,500 rpm/10 min/RT; [15]). Trypomastigotes were then collected and counted in a CASY Cell Counter (Roche-Applied-Science) using the 60 μm capillar. Trypomastigotes, in supplemented assay DMEM, were added to H9c2 cultures in a multiplicity of infection (MOI) = 1 and incubated for 18 hours. Cells were washed once with PBS before incubation of the infected H9c2 monolayer with trypsin (Life-Technologies) to detach cells from the flask. Cells were counted in a CASY Cell Counter (Roche-Applied-Science) using the 150 μm capillar and their density set at 5×10^4^ cells per ml in supplemented assay DMEM.

### Assay for testing susceptibility of *T. cruzi* to compounds

Infected H9c2 were dispensed into 384 well plates (μClear bottom, Poly-Lysine CellCoat^®^ treated surfaces; Greiner Bio-One, Germany) at 50 μl per well using a Multidrop (Perkin-Elmer) liquid handling device. Control wells indicating 100% parasitic growth were left untreated, whereas control wells defining 0% parasitic growth just contained H9c2 uninfected cells. The rest of the plate contained the compounds dispensed according to the dose regimen explained in the previous section. DMSO concentration never exceeded 0.5% in all plate wells, which had no effect on the host cells viability, neither in the parasite replicative growth. DMSO tolerability limit of the assay was found to be 2%. After seeding them, the plates were incubated at 37 °C in a humidified 5% CO_2_ atmosphere for 72 h. Cultures were then fixed and stained by addition of 50 μl of a solution containing 8% formaldehyde and 4 μM Draq5 DNA dye (BioStatus, UK) per well. Plates were kept light-protected and imaged 1 h later (longer times of incubation did not affect the quality of the assay).

### Image acquisition

Plates were imaged in a Perkin-Elmer Opera confocal microscope using a 20X air objective (NA 0.4) and the next acquisition set: a 635 nm laser excitation line and a 690/50 emission detection filter for Draq5 detection (bandwidth 650–700 nm). Five images were collected per each well for reliable statistical analysis. No significant differences were observed in cell or amastigote numbers among images in different locations within wells. The Opera microscope in our facility is coupled to a robotic arm that automatically loads and unloads the plates to be imaged, contributing to an increased throughput when required.

### Image analysis

**Fig 1:**
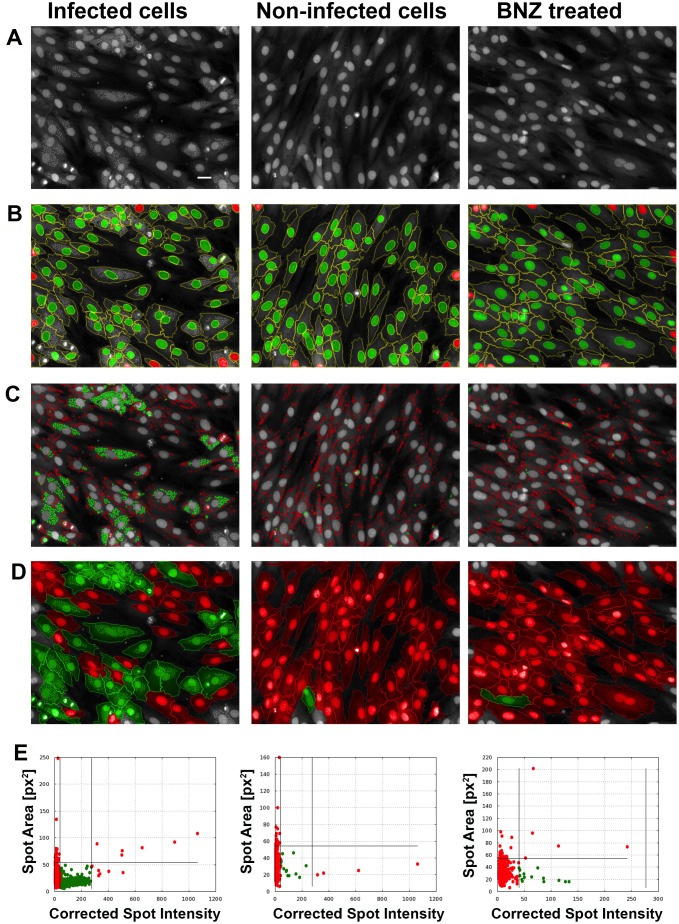
Image analysis development. Representative images of the *T. cruzi* infected control, non-infected control, and 30 μM benznidazole treated infected cells (BNZ) for: (a) raw image acquisition (at 635 nm); (b) nuclei selection (selected in green and discarded in red; as described in Methods) and their cytoplasm boundaries identification (green lines); (c) discrimination of true amastigotes (green) from other non-parasitic cytoplasmic spots (red) amongst all cytoplasmic spots detected; (d) identification of infected cells (those with >1 amastigote inside, green) and non-infected cells (red); (e) scatter plots of all detected spots used to discriminate true (green dots) from false amastigote spots (red dots) based on their area and intensity (‘Spot area’ and ‘Corrected intensity’ thresholds lines are shown; ‘Corrected Intensity’ is expressed in arbitrary units). Bar is 30 μm.

To define presence or absence of *T. cruzi* amastigotes within delimited cell boundaries (cells cytoplasms) the Acapella software (Perkin-Elmer) was used to develop a very selective script based in the algorithms provided by the software´s ‘building blocks’ approach. The script included the following steps (Fig. 1):

- Confocal images acquisition from the Opera microscope (Fig. 1a).
- Identification and selection of the host cells nuclei was based on: nucleus mean intensity (>100 fluorescence intensity counts; arbitrary units, a.u.), nucleus area (>60 and <1000 μm^2^) and morphological features (width and roundness >0.3 a.u.) (Fig. 1b). Cells whose nuclei are touching the border of the image were discarded from further analysis (Fig. 1b, red). Cells whose nuclei are intact but their cytoplasm is partly outside the field of view were included since an analysis of their cell area and amastigote density showed no significant differences with intact cells.
- Cytoplasm region extrapolation of each host cell, based on the nuclei identified in step II. Cytoplasm was extrapolated expanding the area around the nuclei until the fluorescence signal from Draq5 staining becomes equal to the image background (areas with no cells), which marks the edge of the cell. Cell boundaries are shown in Fig. 1b delimited by yellow lines.
- Identification and selection of intracellular spots corresponding with *T. cruzi* amastigotes nuclei present in the previously identified host cells cytoplasmic regions (Fig. 1c). A very inclusive spot detection method was used, which was followed by a very selective method to discriminate true amastigotes (Fig. 1c, green) from other host cells cytoplasmic spots (Fig. 1c, red). It was based on ‘Corrected intensity’ and ‘Spot area’ properties of the identified spots. Further explained below in ‘Results’ section.
- To account the percentage of infected cells, those host cells bearing at least two spots in their cytoplasmic regions were considered infected (Fig. 1d, green); whereas uninfected host cells contained either none or one spot (Fig. 1d, red). It was decided to define as ‘infected cell’ that host cell containing >1 spot as with this criteria the Z´ and signal/background ratio of the assay were improved due to a reduction of the impact of the non-specifically stained background noise. Such unspecific spots that appear in uninfected cells most probably correspond to cytosolic accumulations of host cell RNA.
- A scatter plot was generated from pictures of *T. cruzi* infected cells, non-infected cells, and BNZ treated (30 μM) infected cells positioning each detected spot within them based on their ‘Corrected intensity’ (X axis) and ‘Spot area’ (Y axis) parameters (Fig. 1e). The two ‘Corrected intensity’ thresholds (vertical lines) and the ‘Spot area’ threshold (horizontal line) were determined with the following equation since both spots populations parameters followed a lognormal distribution:

sd(x)=(eσ2−1)e2μ+σ2, over the expected value $E(x)=e^{\mu +\sigma^{2}/2}$

(The method to determine these thresholds is explained in “Image analysis method” section of Results). The spots eventually counted as true amastigotes are shown in green. It is observed that there are remaining putative spots which contribute to a certain ‘noisy’ background in non-infected cells and BNZ treated ones (see corresponding panels in Fig. 1e).

### Data analysis

Three outputs (per well) were provided by the script: (1) number of host cells nuclei (‘Cells’) to determine drug-related cytotoxicity; (2) number of amastigotes per cell (‘Am/Cell’) as infection level measurement; and (3) percentage of infected cells per well (‘%Infected’) as a second infection level output. The assay window (signal to background; S/B) and the Z´ parameter assessment were performed as previously described [29]. To qualify a plate as pass, Z´ values had to be >0.5. The three selected outputs per plate were exported to ActivityBase software (IDBS, USA) for data normalization and fitting to inhibition equation.

The TC_50_ value was provided by the ‘Cells’ output and two IC_50_ values were respectively provided by the ‘Am/Cell’ and ‘%Infected’ outputs. All curves visualization and fitting was carried out with ActivityBase, following the equation by Hill’s 4 parameters being the maximum asymptote between 80 and 100% for all the compounds presented in the article. The selectivity window was calculated for each compound in the plate taking into account their TC_50_ and ‘Am/Cell’ IC_50_ values. Results summarized in Table 1 are referred to at least three independent experiments and in all cases are shown as the mean value ± its standard deviation (± sd).

## Results

### Assay development

Our goal was to develop an assay specifically aimed at the parasitic amastigote stage, since this is the replicative form in the mammalian host. A rat myoblastic cell line (H9c2) was chosen as host cells, since *T. cruzi* pathology is frequently related to its presence in muscle tissue during the chronic stage of the disease [2, 4] and these cells are highly susceptible to infection *in vitro* [30-, 31]. Monolayers of H9c2 myocytes were infected with *T. cruzi* trypomastigotes for 18 h to allow for development of the intracellular amastigote stage. Infection was performed in T-flasks followed by washing to remove non-invasive trypomastigotes present in the culture medium and trypsinization of the cell monolayers. Several times and multiplicities of infection in the flasks, and host cell numbers per well after transfer to plates, were tested in order to define the best possible assay. We observed that 5×10^4^ cells/ml (2,500 cells per well) provides sufficient cell events counts per view field for an statistically robust measurement (Z’ was used as the discriminatory parameter) without compromising the imaging and image analysis times. A MOI = 1 was considered optimal since it provided a ~50% infection level after 18 h incubation in the flask. The total assay timespan that includes 18 h in the T-flasks and 72 h in the plates (~90 h) permits various parasite replication rounds with negligible host cells bursting due to over-infection (cell integrity was monitored for every condition tested using the parameter ‘Cells’ as described below). Cells were then dispensed into 384 well plates (2,500 cells/well) coated with PolyLys (CellCoat^®^ by Greiner-BioOne). At 72 h incubation, confluency of monolayers was found to be 52.15% of the well surface (average of six independent control wells). Cultures were then stained with a DNA dye, Draq5 (Fig. 1). Similarly to previously reported [32], no major differences were seen between visual or automated counting of amastigotes, with a quantification correlation>90% in four independent experiments.

### Image analysis method

**Fig 2:**
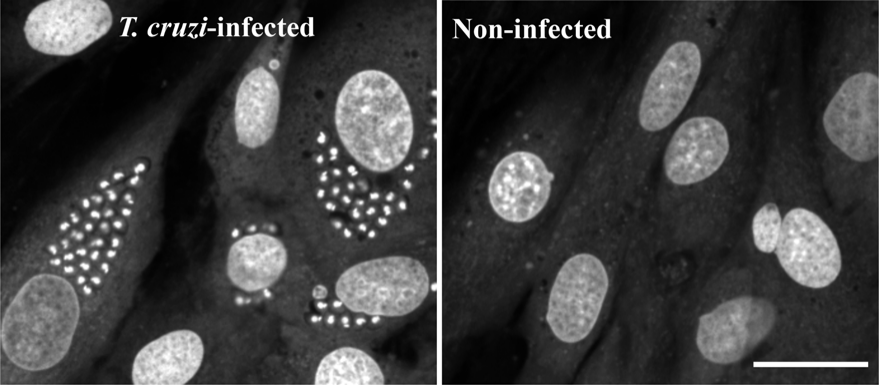
High resolution image of controls. Representative *T. cruzi* infected and uninfected H9c2 pictures at 40X. Kinetoplastid kDNA (circle) and nDNA (bean-like) can be distinguished at this magnification. Bar is 30 μm.

When images were acquired at 40X, it was observed that, in addition to host cell nuclei, both the nuclear DNA (nDNA) and mitochondrial kinetoplast DNA (kDNA) were stained (Fig. 2). Nevertheless, at the magnification rate used for the automated assay (20X) it was not possible to discern between parasitic nDNA or kDNA and a single spot per amastigote was observed (Fig. 1). Draq5 slightly stains the host cells cytoplasm too, which helped to define the surface of the host cell and differentiate it from the highly stained host cell nuclei with the algorithms from the Acapella software (Fig. 1b). The size differences between the host cells and the *T. cruzi* amastigotes nuclei permitted the use of a single fluorescence channel to detect both cell types (Ex/Em = 635/690 nm). For accurate quantification of amastigote spots per cell, two parameters were evaluated by the script: ‘Spot area’ to differentiate between host cells and parasite nuclei with a cut off for parasite spot detection at ~35.5 μm^2^ (~55 px^2^) and a ‘Corrected intensity’ filter with two thresholds to distinguish true parasitic spots, that have higher intensity, from non-parasitic (‘noisy’) cytoplasmic spots per cell (Fig. 1c; see below for details). These thresholds varied slightly per run of plates and were therefore defined considering the control wells of each one prior to the performance of analysis.

**Fig 3:**
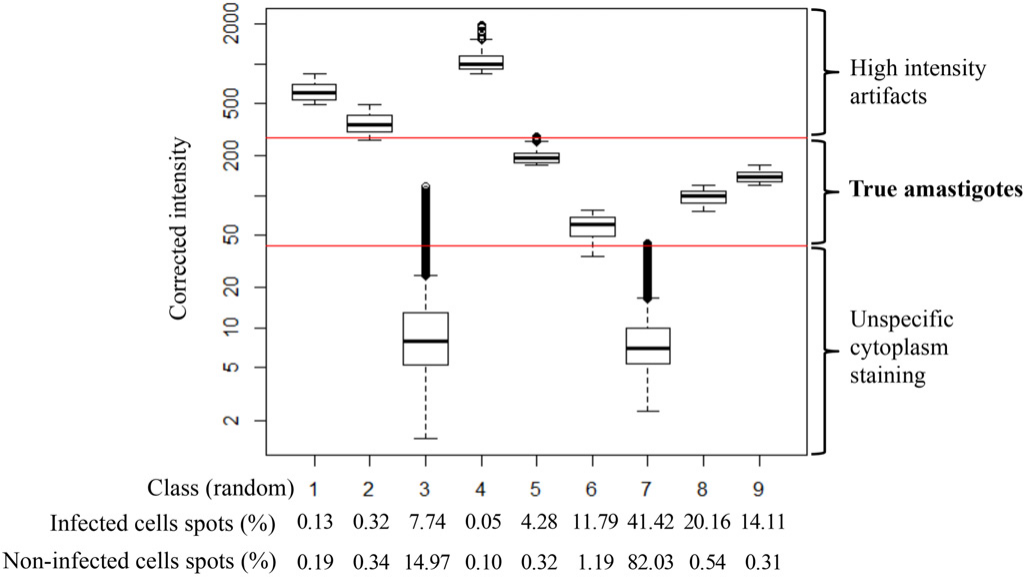
Discrimination of spots using ‘Corrected intensity’ as parameter. A total of 1.18×10^6^ spots were acquired from infected and non-infected control wells (five fields per well from 96 wells for each control) from 6 independent plates each. Spots were distributed in 9 groups and classified accordingly to their ‘Corrected intensity’ parameter based on the Hartigan-Wong algorithm [34]. The three parametric regions (low, intermediate and high) are indicated by red threshold lines in the box plot. Percentage of spots from infected and non-infected controls in each of the nine classes is shown below

**Fig 4:**
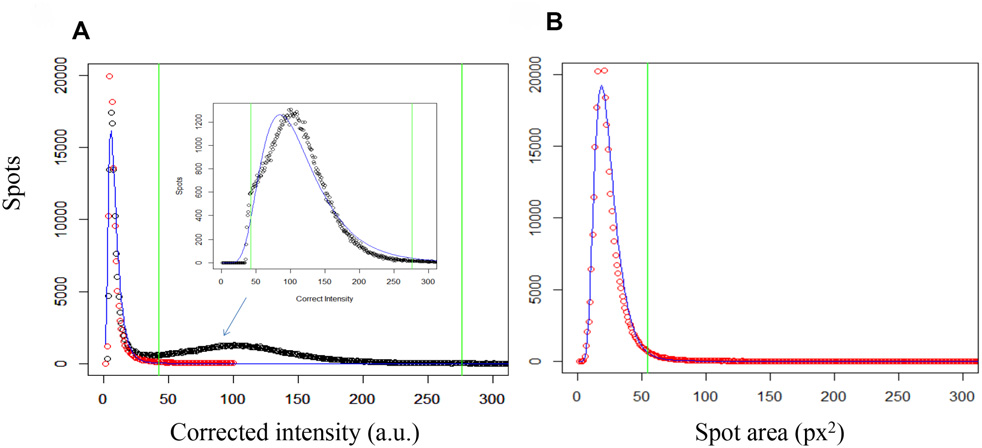
Determination of thresholds. ‘True amastigote’ discrimination thresholds based on their (a) ‘Corrected intensity’, and (b) ‘Spot area’ parameters were defined from the spots populations within both, infected and non-infected control wells (lognormal adjustments, blue lines; lower and upper ‘Corrected intensity’ thresholds (respectively at 48 and 274 a.u.), and the upper ‘Spot area’ cut off (51 px^2^), green lines). Inset in panel (a) zooms in at the spots distribution in the intermediate ‘Corrected intensity’ region (true amastigotes).

A high number of non-parasitic stained cytoplasmic spots were detected as a consequence of non-specific dye staining (see Fig. 1b and [18, 33]). In order to find a method to best discriminate true amastigotes from other non-parasitic cytoplasmic spots, all fields from control infected and control non-infected cells within six independent plates were taken. Those images were processed using Acapella software with the sensitivity for spot detection set at maximum. The spots retrieved this way (1.18×10^6^) were classified based on their ‘Texture’, ‘Corrected intensity’ and ‘Morphology’ properties into nine classes by an initial clustering method based on the Hartigan-Wong algorithm [34] and processed with the statistical analysis software ‘R’ [35] (Fig. 3). This analysis revealed that ~95% of the spots population in control non-infected wells were accumulated in two of the nine classes (classes 3 and 7 in Fig. 3). A further graphical analysis of the spots populations according to their ‘Corrected intensity’ parameter revealed three discriminating regions for that property and it was observed that those spots that fell into classes 3 and 7 (note they meant a ~95% of the spots retrieved from non-infected controls) belonged to the low ‘Corrected intensity’ region, which therefore determined the spots non-specifically stained by Draq5. In contrast, a significant percentage of the spots found in *T. cruzi*-infected control wells populated the intermediate ‘Corrected intensity’ region identifying this as the true amastigotes region. The third high intensity region included scarce artifacts of the staining procedure with very high corrected intensity values (Fig. 3). Thus, based on this analysis the ‘Corrected intensity’ condition from the Acapella script was selected to discriminate true from false amastigotes. In order to determine the limits of that medium ‘Corrected intensity’ region that contained the true amastigotes a lower and an upper threshold were set. The lower cut off limit was set by adjusting all non-infected control spots population ‘Corrected intensity’ (this population mainly fell into the low ‘Corrected intensity’ region) to a lognormal distribution and establishing the threshold at 6 standard deviations (sd; Fig. 4a). Then, the upper threshold was determined by adjusting to a lognormal distribution all the spots in infected control wells whose ‘Corrected intensity’ was above the low limit calculated before, plus 3 sd taken over the expected value (see equation in Image analysis section of Methods; Fig. 4a). Lastly, to further contribute to best discriminate true from false amastigotes, an upper threshold for the ‘Spot area’ parameter was also set (Fig. 4b). To define it, all infected control spots that fell between the lower and upper ‘Corrected intensity’ thresholds explained above (i.e. true amastigotes) were adjusted to a lognormal distribution and their upper ‘Spot area’ threshold was set at 3 sd over the expected value, thus excluding from a further analysis those spots above such threshold (Fig. 4b). The final established criteria for automated identification of true amastigotes selected spots consisted on intermediate ‘Corrected intensity’ between the pre-set thresholds (>48 and <274 a.u.) and a ‘Spot area’ size below a cut off of 51 px^2^ (Fig. 1e and Fig. 4). These values, assigned during the statistical analysis procedure that was followed to build the script were empirically customized for each corresponding run (or assay day) by statistical analysis of the spots from all control wells in the run.

**Fig 5:**
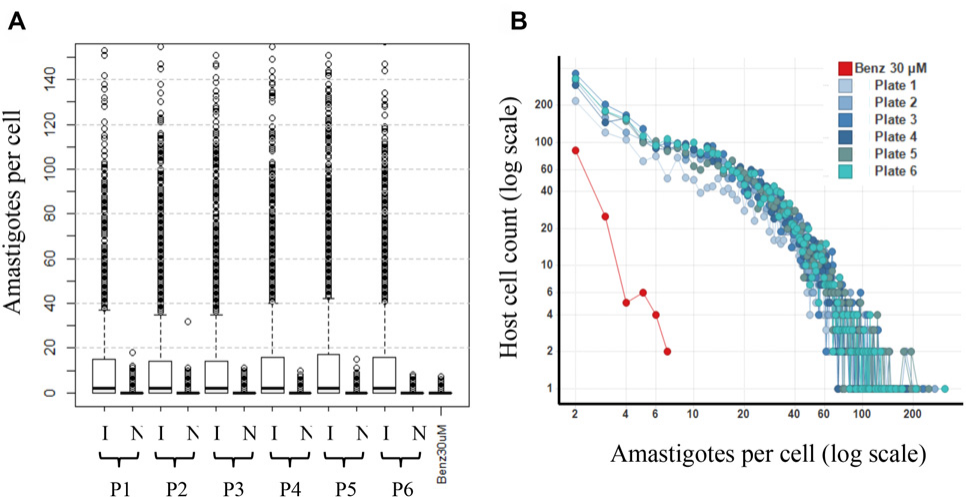
Individual analysis. Controls for *T. cruzi* infected (I) and non-infected (N) cells of six independent plates (P1–P6) are represented: (a) box plot of the number of amastigotes per cell for each control: I (infected untreated cells) and N (non-infected cells) (16 wells of each per plate), and BNZ treated infected cells (30 μM; 16 wells in one plate) is shown. The distribution of the populations is not normal, so the median value per plate (black line) is shown. The second (white box under median), third (white box over media) and forth (dashed line) quartiles and population outliers (dots) are represented. (b) Scatter plot correlating the number of host cells to the number of amastigotes per cell. Infected cells control wells of each plate (blue lines) and BNZ treated infected cells plate (red line).

With the algorithms available for the script design, the outputs provided a mean readout per well. To test the reliability of the mean readout and to inform on the infection homogeneity achieved, a cell-by-cell spots population analytic approach was run on both controls (infected and non-infected) in six assay plates made in different days. The analysis showed a very homogenous performance independently of the plate, where the median of amastigotes per cell in control infected was ~10 amastigotes per host cell in all cases, in contrast with the very seldom control uninfected cells that contained >1 putative parasites (population outliers in Fig. 5a). The number of amastigotes per cell (median) in control uninfected cells was very similar to that found in BNZ treated infected cells (Fig. 5a). The analysis was used to categorize the distribution of the number of amastigotes occurring per individual cell. This showed that a very large percentage of the ~50% of infected cells contained over 8 parasites; whereas in BNZ-treated infected cells the few hosts that contained amastigotes were below that number (Fig. 5b).

### Assay performance

Compound inhibition of *T. cruzi* replication was primarily assessed as the reduction of the mean amastigote number per cell (‘Am/Cell’), as well as by a decrease of the percentage of infected cells per well (‘%Infected’). Since parasite number reduction could potentially be due to a drug-related cytotoxic effect on the host cells, the mean host cells nuclei number per well (‘Cells’) was considered in the analysis procedure. The reference value that indicated a 100% of live host cells was always established upon non-treated control infected wells. Five image fields were taken per well, which provided a statistically significant Z’ values for the assay. In all cases, at the assay readout end point (~90 h post-infection; 72 h after addition of compounds) similar host cells number was seen per well in infected control (mean ± sd; 353.92 ± 24.22) and non-infected control wells (mean ± sd; 334.13 ± 23.35), which indicated that host cell lysis was not significantly occurring in the 90 h infection time span.

A reduction in the ‘Am/Cell’ and in the percentage of infected cells per well or ‘%Infected’ measurement in compound-exposed wells, in comparison with the values obtained for the non-treated infected control wells, both yielded a quantitative reading of *T. cruzi* growth inhibition after 72 h of drug exposure. In untreated infected control wells, the average number of amastigotes per cell was 10.19 ± 1.22, which contrasted with the 0.39 ± 0.08 in non-infected control wells. We established non-infected control wells as 100% of parasite growth inhibition, which defined a 25X signal-to-background window. Under these conditions, the ‘Am/Cell’ output presented acceptable reproducibility with a Z´ of 0.6 (calculated from 16 control wells of three different plates from three independent experiments). The third output, ‘% Infected’, provided a complementary reading of the anti-*T. cruzi* activity of the compounds and served as well as an assay performance inner control. In all cases, the ‘% Infected’ was above 50% (mean ± sd; 52.44 ± 3.03) in the non-treated infected control (Fig. 1d), and contrasted with that of non-infected control wells (8.72 ±.88). These data offered a signal to background window (S/B) ~6X, which contributed to a robust Z´ parameter also for this output, that was calculated to be 0.66. As quality control for every assay, a requirement for a Z’>0.5 in each of the three main parameters (‘Cell’, ‘Am/cell’ and ‘% Infected’) was considered.

At present, 162 replicate plates of the assay have been performed over a period of 10 months with remarkable reproducibility. The Z’ values of the parameter ‘Am/Cell’ remains consistently >0.5 with an average value of 0.58.

### Validation

To validate the assay a list of compounds with known anti-trypanosomatid activity was assayed at 11 serially diluted concentrations at least in three independent experiments (Table 1). Most of the compounds in the list had been previously tested against a range of *T. cruzi* strains either by image-based [17–18] or other *in vitro* assays [15–16, 19, 26–27], including the primary assay of the recent anti-*T. cruzi* high throughput screening campaign (HTS) performed in GSK Tres Cantos facility (Spain; Peña et al., manuscript in preparation). Our assay succeeded in identifying all the anti-*T. cruzi* compounds, and the dose-response analysis made assigned TC_50_ and IC_50_ values that were in concordance with those previously reported [15–17, 19, 26–27]. Agreement was best with those previously reported results where compounds were tested against a Tulahuen parasite strain [15, 17, 19, 27], particularly when looking at the Am/Cell assay output. A comparison between our retrieved dataset and the previously one reported by Engel et al. anti-*T. cruzi* image-based assay [17], also showed a good agreement despite the use of different parasite strains and host cell lines in both assays. Looking at Chagas disease standards of care drugs, BNZ and NFX, the results obtained here are very similar to those previously published for the first, but a certain disagreement was observed for NFX IC_50_ [25], which is probably due to the use of epimasgote forms to assess anti-parasitic drug performance [25].

**Fig 6:**
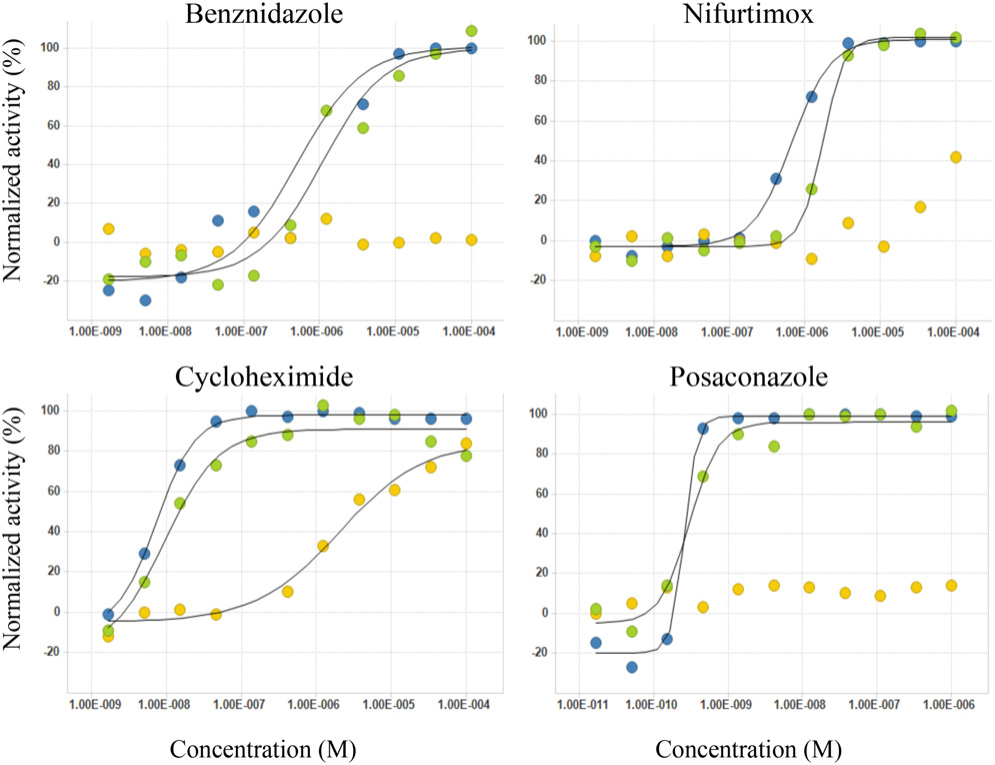
Drugs dose-response curves. Representative curves of three independent experiments of BNZ, NFX, posaconazole and cyclohexymide normalized activity (%) against *T. cruzi* amastigotes per compound concentration (M) for each of the three assay outputs: ‘Am/Cell’ (blue), ‘%Infected’ (green) were normalized as described in [15]; ‘Cells’ (cytotoxic impact of the drugs; yellow) is expressed as percentage of live cells, considering non-treated *T. cruzi* infected wells as 100%.

The response curves of two current drugs used in the clinic for treatment of Chagas disease (BNZ and NFX), posaconazole (recently tested in clinical trials with negative results) and cycloheximide (second compound in potency against *T. cruzi* amongst the list tested are shown to illustrate the versatility of the image-based approach that provides the toxicity to the host cells and two anti-parasitic response outputs, amastigotes per cell and percentage of infected cells, in a single experiment (Fig. 6).

## Discussion

There is a pressing need for new compounds to treat Chagas disease. With the perspective to accelerate pre-clinical screening of putative anti-Chagas compounds, we have developed an image-based phenotypic whole cell assay that identifies anti-*T. cruzi* hits specifically targeted against the intracellular amastigote stage. This is the replicative form in the mammalian host and therefore is considered the major target for anti-*T. cruzi* future drugs. Imaging assays anti-*T. cruzi* and an imaging-based screening have been reported before with successful results [11, 17]. Our assay provides novel features that constitute important advantages for the performance of HTS campaigns. In addition to the parasite growth inhibition readout, this imaging-based assay provides a cytotoxic readout, therefore providing in a single experiment the drugs specific activity against the parasite versus the host (selectivity index). High statistically robust discriminating power (better Z’ values) and strong assay reproducibility, in addition to the highly reproducible image analysis method together with the robotic automation of the plates’ loading-unloading process guarantee medium-to-high throughput capabilities, therefore providing a versatile assay that could either be used as a primary screening tool (single dose) as well as a secondary hit qualifier assay (dose response).

Along the development process, a fundamental aspect was the election of the host cell line to support parasite replication. We chose the myoblastic H9c2, which had previously been used to characterize the *T. cruzi* infectious process [30–31]. H9c2 cells provided percentages of infection above 50% in untreated control wells at MOI of 1 (Fig. 1d). These conditions resulted in homogeneous infections where the rate of infected to non-infected cells was similar in all areas of the well. This factor was crucial for the reproducibility of the parameters determined in the assay, ratio of amastigotes per cell and percentage infected cells. Previous attempts to use NIH3T3 fibroblasts as host cell resulted in non-homogenous infections that provided low reproducibility in the parameters determined.

While H9c2 are easy to culture and allowed very reproducible experiments and a robust assay performance, they have a large size (~100 μm), which accounted for a low production rate. To overcome this problem, H9c2 cells were cultured in roller, which resulted in a highly increased yield. A single roller supplied cells to seed at least seven T225 flasks at the assay day. Given that host cells for three 384 well plates are obtained per each infected T225, the achieved throughput may reach >20 plates per roller. This translates into 7,000 single dose or 640 dose-response evaluations plus their corresponding controls per roller, conditions that allow for high throughput screening using this method. In our facility, four roller flasks per week were typically used, allowing testing of approximately 35,000 compounds and the processing of 80–100 plates.

It was our objective to develop a reliable and easy to standardize assay. With that in mind, the pre-infection of the cells was made in flasks instead of in plates allowing homogenous infected cells batches and simplified the programmed washing steps. The use of a single stain, Draq5, for host and parasite cells also simplified the procedure. DAPI has previously been used as a single stain for host cells and *T. cruzi* [17], however, Draq5 dye provided a faint stain of the host cell’s cytoplasmic area, which facilitated the detection of this region in the analysis [18, 33]. Furthermore, as Draq5 can be added together with the formaldehyde fixing solution and no washing steps are required thereafter, the fixation and staining of the assay plates was made in a single step, saving time and avoiding washing steps that may have affected the monolayers. Although the use of a single dye carried the disadvantage of certain unspecific staining of non-parasitic cytoplasmic spots, that was circumvented with a very selective script analysis. Avoiding the use of two different dyes also improved the speed of image acquisition and analysis. An additional advantage of the use of a DNA stain is the possibility to use any strain or clinical isolate of *T. cruzi* that can be cultured *in vitro*, upon assay adaptation to its particular characteristics, since it does not require transgenic lines of parasite expressing reporters.

The assay was validated against a series of compounds that had been previously reported to have anti-trypanosomatid activity [15, 17, 19–28]. Despite the expected variability in the results due to the different methods and strains used in different laboratories, our retrieved results mostly agree with the previously available data, being the correlation particularly good with those references that had used the Tulahuen strain [15, 19, 27].

High-content screening is nowadays considered a key technology in the drug development process by the pharmaceutical industry. Nonetheless, several aspects mainly linked to the large volume information generated and the complications associated to data integrity, storage and processing still need to be improved [13]. Our assay has been designed to allow high level data integrity and data tracking featured upon barcode identified plates. It is therefore essential that the readouts can be followed by data management software like the ActivityBase suite (IDBS, USA). With that in mind, three simple outputs per well were selected to provide the needed information to determine whether a compound was or not toxic to the host cells; whether it actively inhibited the parasite growth; and to complement such information, whether the infectivity ratio was constant intra- and inter-plates, which also served as an assay quality control readout. Another important aspect in the development of the assay was the processing time required for both imaging and image-analysis, as these constitute potential throughput bottlenecks. The use of a single laser channel to detect host cells and parasites (Draq5 nucleic acid dye) contributed to reduce the imaging time, performing just one exposure over the five fields acquired per well. Once the plate was inside the Opera microscope, it took 1 h to perform the image acquisition and analysis. But if required, the Perkin-Elmer Opera device and its associated Acapella software allow independent image acquisition and analysis, so the software can be set to run offline upon pre-taken stored images, circumventing the time-consuming computing issue.

The great versatility and reproducibility afforded by this image-based approach may provide the basis for further advancements in the near future, like expanding the panel of *T. cruzi* strains for testing to allow a wider coverage of the wide phylogenetic spectrum of the parasite. Other potential upgrade chances may lie on the development of an automated assay to discern between cidal and static compounds [15], which would provide further tools for the selection of early hits. Imaging assays rely on the fast advancing optical and computing technologies and definitely stand as a very powerful and adaptable tool in the drug discovery process.
